# Surgical approach and treatment for the orifice of a pyriform sinus fistula: a case report and literature review

**DOI:** 10.1093/jscr/rjaa170

**Published:** 2020-06-18

**Authors:** Yukihiro Tatekawa

**Affiliations:** Department of Pediatric Surgery, Saku Central Hospital Advanced Care Center, Nagano 385-0051, Japan

**Keywords:** pyriform sinus fistula, endoscope, open-neck surgery

## Abstract

A 4-year-old girl underwent an open-neck surgical procedure for a recurrent pyriform sinus fistula (PSF). A catheter could not be inserted through the endoscope into the opening of the fistula. An open-neck surgical procedure with coring out of the fistula stained with a dye revealed that the fistula was missed near the upper lobe of the left thyroid. A guide wire was successfully inserted via the endoscope into the fistula, and the wire was gripped with forceps under fluoroscopic guidance and removed. As a modification of the surgical approach and treatment for the orifice of the PSF, a catheter was exchanged through the guide wire, and a nylon thread was passed into the catheter. The tip of the nylon thread from the oral side was fastened and fixed to a gauze ball. After removing the nylon thread, the orifice of the sinus fistula was recognized and sutured. She was discharged uneventfully and has done well without a postoperative recurrence for 12 months.

## INTRODUCTION

Management of a pyriform sinus fistula (PSF) has included an open-neck surgical procedure, laryngoscopic suture closure and endoscopic/laryngoscopic surgery with chemocauterization, electrocauterization, laser cauterization or application of surgical glue [1–8]. Regardless of the method performed, it is important to close the orifice of the PSF.

## CASE REPORT

**Figure 1:**
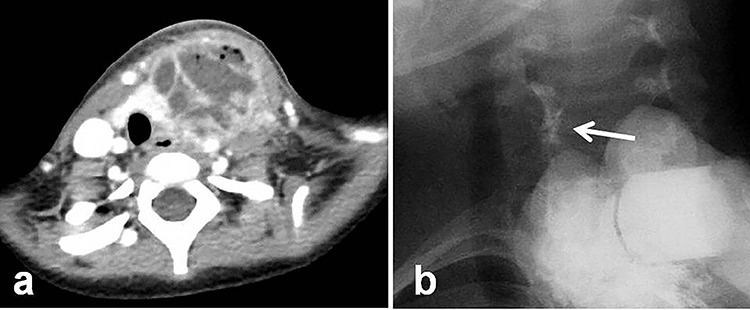
Radiologic examinations. (**a**) Enhanced computed tomography showing a huge abscess. (**b**) At the second hospitalization, direct fistulography from the wound showed an obvious fistula from the abscess cavity to the left pyriform sinus (white arrow: fistula).

**Figure 2:**
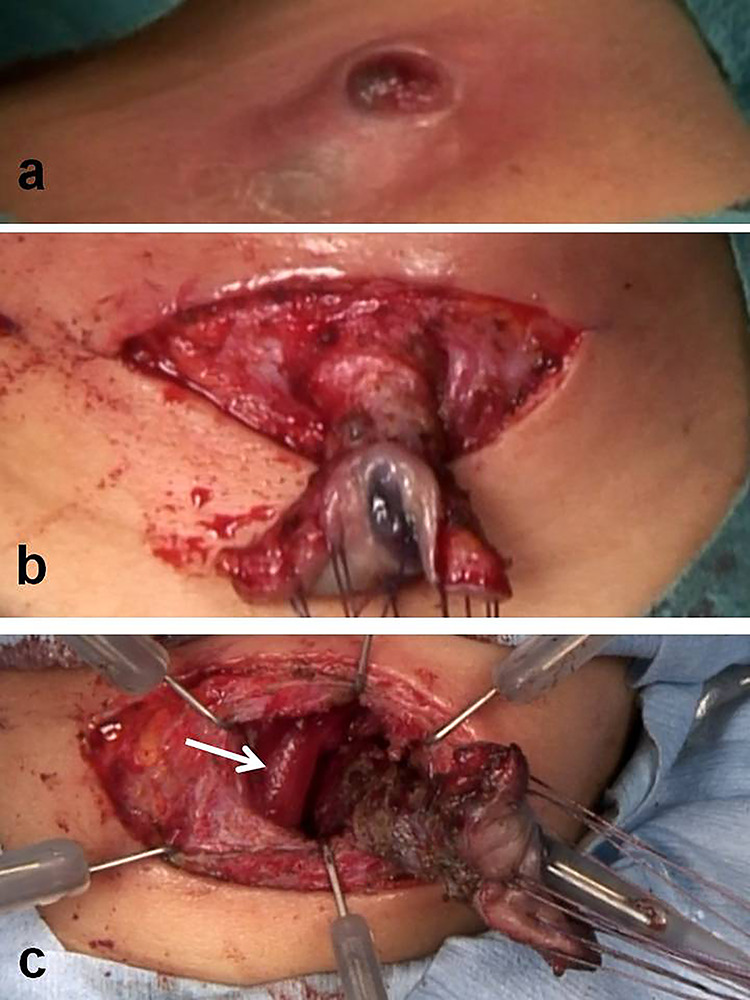
Intraoperative findings with open-neck sugary. (**a**) At the third hospitalization, a recurrent pyriform sinus fistula with abscess was demonstrated. (**b**) After direct injection of contrast medium and indigo carmine into the abscess cavity, an open-neck surgical procedure was done with coring out of the fistula stained with a dye. (**c**) A fistula being missed near the upper lobe of the left thyroid and the scar tissue with abscess (white arrow**;** left thyroid).

**Figure 3:**
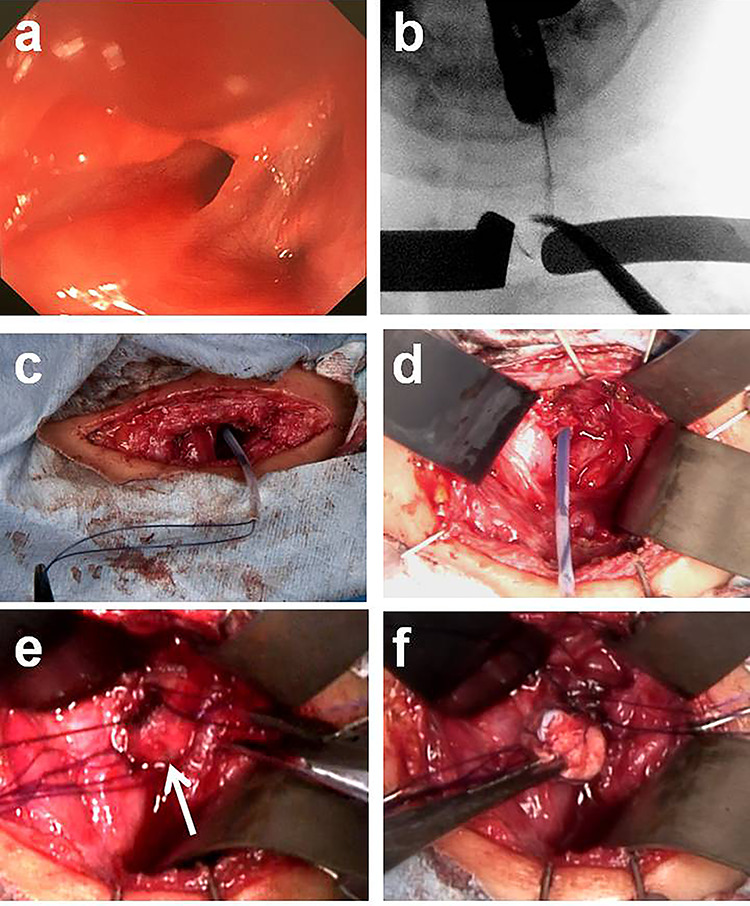
Intraoperative findings with treatment of a pyriform sinus fistula. (**a**) Orifice of the sinus fistula in the left pyriform sinus. (**b**) A guide wire was successfully inserted through the endoscope into the fistula, and the wire was gripped with forceps under fluoroscopic guidance. (**c** and **d**) A catheter was exchanged through the guide wire and a nylon thread was passed into the catheter. (**e** and **f**) After removing the nylon thread, the gauze ball was also removed.

**Figure 4:**
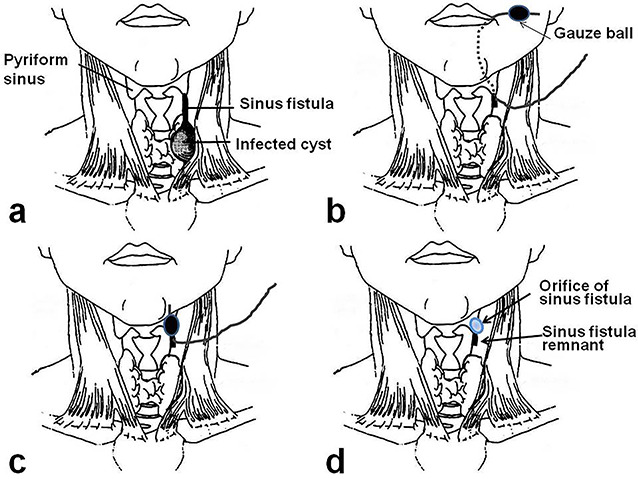
Scheme of improvement of the surgical approach and treatment for the orifice of the pyriform sinus fistula [9]. (**a**) Pyriform sinus fistula. (**b**) After a nylon thread was passed into the catheter in the pyriform sinus fistula, the tip of the nylon thread from the oral side was fastened and fixed to a gauze ball. (**c**) After removing the nylon thread, the gauze ball was packed at the orifice of the pyriform sinus. (**d**) After the gauze ball was removed, the orifice of the sinus fistula was recognized.

**Figure 5:**
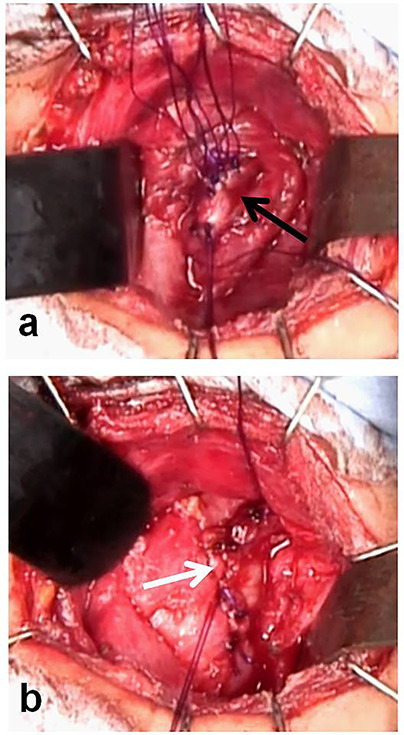
Closure of the orifice of the pyriform sinus fistula. (**a**) Closure of the thyropharyngeal part of the inferior constrictor of the pharynx (black arrow: the thropharyngeal part). (**b**) Closure of the cricopharyngeal part of the inferior constrictor of the pharynx (white arrow: the cricopharyngeal part).

A 4-year-old girl was admitted with left neck swelling. The physical findings showed a huge mass with erythema in the left neck. Enhanced computed tomography showed a large abscess (Fig. 1a). After incision and drainage, a barium swallow examination and direct fistulography from the wound showed no fistula. Five months later, she was hospitalized for the second time for left neck swelling. After incision and drainage, a direct fistulography from the wound showed a fistula from the abscess cavity to the left pyriform sinus (Fig. 1b). She was diagnosed with a pyriform sinus fistula (PSF). She was followed in the outpatient clinic with serial ultrasonograms for elective surgery, but after 5 months, she was readmitted with recurrent left neck swelling before surgery was scheduled. Thus, she underwent an open-neck surgical procedure for recurrent PSF. First, the catheter was inserted into the opening of the fistula through an endoscope and contrast medium was injected, which did not reveal a fistula from the left pyriform sinus to the abscess cavity. Next, after direct injection of contrast medium and indigo carmine into the abscess cavity, an open-neck surgical procedure was performed with coring out of the stained fistula, but a fistula was overlooked near the upper lobe of the left thyroid, and the scar tissue with abscess was removed (Fig. 2a–c). Finally, a guide wire was successfully inserted through the endoscope into the fistula; the wire was gripped with forceps under fluoroscopic guidance and removed (Fig. 3a and b). As a modification of the surgical approach and treatment for the orifice of the PSF, a catheter was exchanged through the guide wire, and a nylon thread was passed into the catheter (Fig. 3c and d). The tip of the nylon thread from the oral side was fastened and fixed to a gauze ball (Fig. 4a and b) [9]. After removing the nylon thread, the gauze ball was also removed and the orifice of the PSF was noted (Figs 3e, f and 4c, d). The orifice of the PSF was doubly closed with absorbable sutures of the thropharyngeal and cricopharyngeal parts of the inferior constrictor of the pharynx (Fig. 5). She was discharged uneventfully and doing well without a recurrence 12 months postoperatively.

## DISCUSSION

Complete excision of a PFS is the common treatment. Recently, as a minimally invasive surgery for PSF, endoscopic surgery for the opening of the fistula has been reported [1, 2]. In endoscopic surgery, closure of the opening of the fistula is sutured through direct laryngoscopy [1, 2]. This method is useful to try initially for a PSF, but the risk of a recurrent infection from the residual fistula tract is a limitation. Endoscopic surgery is difficult for the treatment of PSFs in children because the working space is small and narrow [2]. Furthermore, endoscopic surgery requires machine settings and techniques. In chemocauterization of the fistula tract, trichloroacetic acid or silver nitrate has been used; however, albuminoid degeneration in tissues causes scar formation [3, 4]. Trichloroacetic acid or silver nitrate might affect the recurrent laryngeal nerve, and chemocauterization methods require repeated procedures [1]. Sealing materials, such as Glubran 2 or a combination of Tissucol and Deflux, have also been used during endoscopic surgery [5, 6]. These methods are new procedures and necessitate long-term follow-up for recurrences and complications. Finally, electrocauterization or laser cauterization have been performed [7, 8], but these methods carry the risk of pyriform sinus mucosa penetration [1].

Many reports have described recurrences and complications in open-neck surgery [2]. Fistula resection has widely been accepted, but it is sometimes difficult to identify the fistula owing to severe adhesions associated with recurrent infections or repetitive drainage treatment [2]. In the present case, a fistula was missed near the upper lobe of the left thyroid, and scar tissue was removed with the abscess. A guide wire was inserted through the endoscope into the fistula with difficulty, and the wire was gripped with forceps under fluoroscopic guidance and removed. Using this method, the route of the fistula was recognized. The route of the fistula was present at the deep site of the inferior constrictor of the pharynx. It is important to prevent recurrent infections and the risk of recurrent laryngeal nerve injury. With improvements in the surgical approach and treatment for the orifice of the PSF, a catheter was exchanged through the guidewire, and a nylon thread was passed into the catheter. The tip of nylon thread from the oral side was fastened and fixed to a gauze ball. After removing the nylon thread, the gauze ball was also removed, and the orifice of the sinus fistula was closed with precision. Regardless of the method used, recognition of the orifice of the sinus fistula is indispensable to complete the surgery. It is important to recognize the orifice of the fistula by endoscope or a contrast swallow study/direct fistulography from the wound. It is preferred to postpone the surgery until the orifice of the fistula is recognized.

PSF manifests in neonatal and young children, but there are few case reports about PSF in adult patients [10]. There are no reports regarding the carcinogenic risk in PSF, but the coexistence of thyroid cancer in an adult with PSF has been reported [10]. It is necessary to arrange for long-term follow-up surveillance for thyroid cancer after open-neck surgery or endoscopic cauterization for PSF.

## CONFLICT OF INTEREST STATEMENT

The author has no conflicts of interest. The author alone is responsible for the content and writing of this manuscript.

## FUNDING

No competing financial interests or funding exists in connection with this manuscript.
